# Prenatal ultrasound phenotype of fetuses with recurrent 1q21.1 deletion and duplication syndrome

**DOI:** 10.3389/fped.2024.1504122

**Published:** 2025-01-07

**Authors:** Fengyang Wang, Huijuan Peng, Guiyu Lou, Yanxin Ren, Shixiu Liao

**Affiliations:** ^1^ Henan Provincial Institute of Medical Genetics, Henan Provincial People’s Hospital, People’s Hospital of Zhengzhou University, Zhengzhou, China; ^2^ Department of Ultrasonography, Henan Provincial People’s Hospital, People’s Hospital of Zhengzhou University, Zhengzhou, China

**Keywords:** 1q21.1 deletion syndrome, 1q21.1 duplication syndrome, prenatal ultrasound phenotype, array CGH, CNV-seq

## Abstract

**Objective:**

Our study aimed to collect fetuses with recurrent 1q21.1 deletion or duplication syndrome for systematic clinical phenotype analysis to further delineate the intrauterine phenotype features of the two reciprocal syndromes.

**Methods:**

Prenatal samples, including amniotic fluid and chorionic villus samples, were obtained by amniocentesis and chorionic villus sampling at our center, respectively. In total, 43 fetuses were diagnosed with recurrent 1q21.1 deletion or duplication syndrome via array comparative genomic hybridization (array CGH) or copy number variation sequencing (CNV-seq). Prenatal clinical data, pregnancy outcomes, and individual conditions after birth were collected.

**Results:**

In total, 20 fetuses were diagnosed with 1q21.1 deletion syndrome, and 11 had abnormal ultrasound findings. The most common ultrasound features were renal anomalies, musculoskeletal abnormalities, and increased NT. Other less common ultrasound findings encompassed neurologic abnormalities, cardiovascular defects, absence of the gallbladder, intrauterine growth retardation, and cervical cystic hygroma. On the other hand, 23 fetuses had reciprocal 1q21.1 duplication syndrome, 11 of which had abnormal ultrasound findings, mainly nasal bone abnormalities, cardiovascular defects, increased NT, and neurologic abnormalities.

**Conclusions:**

Our case study suggested that the prenatal clinical phenotypes of the recurrent 1q21.1 deletion syndrome and reciprocal duplication syndrome fetuses were highly diverse with incomplete penetrance. Additionally, our findings should expand the intrauterine phenotype associated with the recurrent 1q21.1 region by a series of prenatal ultrasonic anomalies in this work that were described for the first time, which might broaden knowledge of the genotype and phenotype correlation.

## Introduction

The chromosomal distal 1q21.1 recurrent region is widely recognized for its reciprocal 1q21.1 deletion syndrome (OMIM612474) and 1q21.1 duplication syndrome (OMIM612475), which spans 817 kb of a unique DNA sequence (chr1:146577486–147394506, hg19) and includes at least 7 genes. The mechanism of deletion and duplication in the chromosomal 1q21.1 recurrent region is most likely due to the low copy repeat cluster that can mediate genomic rearrangements (1, 2). Based on the chromosomal breakpoints BP1 to BP4, the 1q21.1 band is divided into distinct proximal region (chr1:145386507–145748064, hg19) and distal region (chr1:146577486–147394506, hg19) that cluster CNVs into two classes. Class I deletions and duplications occur between BP3 and BP4 involving only the distal 1q21.1 region, while class II deletions and duplications occur between BP1/BP2 and BP4 containing both the proximal and distal region (1).

To date, the clinical phenotypic features of 1q21.1 deletion or duplication syndrome have been well-defined with incomplete penetrance and variable expressivity (3–5). Individuals with 1q21.1 deletion syndrome manifested a broad spectrum of nonspecific features, such as developmental delay, including gross motor, fine motor and speech delay; mild to moderate dysmorphic facial features, including eye anomalies, large ears with deficient helices, microretrognathia, bulbous nose, dental deformities and cleft lip/palate; congenital heart defects, including atrial septal defects, ventricular septal defects, pulmonary valve stenosis, patent ductus arteriosus, patent foramen ovale, ascending aorta dilatation and bicuspid valve; genitourinary abnormalities, especially renal anomalies; psychiatric and behavioral conditions, such as schizophrenia, ADHD, ataxia, disruptive behavior, and behavioral disorders; neurologic abnormalities, including seizures, microcephaly, intellectual disability, learning difficulties, and hypotonia; growth abnormalities, including short stature; skeletal abnormalities, including polydactyly and clinodactyly, syndactyly, scoliosis, joint laxity and nail dystrophy; and other clinical phenotype features, including failure to thrive, constipation and gastroesophageal reflux disease (1–3, 5–9).

On the other hand, 1q21.1 duplication syndrome shares clinical features similar to reciprocal deletion syndrome, including developmental delay (speech and gross motor delays, fine motor delay), dysmorphic facial features (eye abnormalities, prominent forehead, broad nasal bridge, long philtrum, low-set ears, cleft palate), cardiovascular anomalies (patent ductus arteriosus, patent foramen ovale, atrial septal defects, carotid artery stenosis, tricuspid regurgitation, enlarged right atrium, aortic arch hypoplasia), psychiatric and behavioral conditions (ADHD, autism spectrum disorder, abnormalities of gait/agility, mood disorder/social or behavioral anomalies), and neurologic abnormalities (seizures, macrocephaly, brain anomalies, hypotonia). Other clinical phenotypes include scoliosis gastric ulcers. Although the clinical phenotypes were similar, the deletions seemed to be more strongly associated with renal anomalies, skeletal abnormalities, and microcephaly. However, the duplications were more strongly associated with autism spectrum disorder and macrocephaly (1–4, 6, 7, 9–11).

Conversely, unlike the well-characterized postnatal clinical phenotype, limited information has been collected on the intrauterine phenotype features of 1q21.1 deletion and duplication syndrome. The prenatal diagnosis of 1q21.1 deletion and duplication syndrome remain challenging. To better understand the relationship between genotype and prenatal phenotype features, we examined the prenatal clinical phenotype of 20 fetuses with 1q21.1 deletion syndrome and 23 fetuses with reciprocal duplication syndrome. The results were compared with the fetuses reported in the literature to achieve a comprehensive overview of the intrauterine phenotypes of 1q21.1 recurrent region. To our knowledge, this is the largest prenatal study of recurrent 1q21.1 deletion and duplication syndrome involving systematic clinical analysis.

## Methods

### Clinical data

We retrospectively reviewed 43 fetuses with recurrent 1q21.1 deletion and duplication syndrome from May 2020–March 2024 who came to our center for genetic counseling. Maternal age at diagnosis ranged from 17–41 years, with a mean age of 30 years. The mean gestational age at diagnosis was 20 years, ranging from 11–30 weeks (Tables 1, 2). All the pregnant women were offered serological screening during the second trimester and routine ultrasound screening during the gestation period. The main indications for prenatal diagnosis include abnormal ultrasound findings, advanced maternal age, high risks of T21 and T18, and other reasons for pregnancy (Tables 1, 2). Follow-up data on pregnancy outcome, childbirth, birth weight, parental phenotype, age at verbal language and walking, growth, and development, intelligence, communication and social interaction were collected.

**Table 1:** Clinical data of all the reported fetuses with 1q21.1 microdeletion syndrome.

Case	Maternal age (years)	Gestational weeks	Indications for prenatal diagnosis	CNV results	Size (Mb)	Fetal karyotype	Inheritance	Pregnancy outcome
Present cases								
1	17	11 + 4	Increased NT (5.0 mm), abnormal ossification of fetal limb at 11w4d; cervical cystic hygroma, absence of fetal humerus, abnormal morphology of the radius, ulna and hand at 16w1d	arr[hg19] 1q21.1q21.2 (146507518–147824207) × 1	1.32	46, XX	Unknown	TOP
2	24	24 + 3	Ventricular septal defect, coarctation of aortic arch	seq[hg19] del(1)(q21.1q21.2)chr1:g.146500000_147840000del	1.34	46, XY	Unknown	TOP
3	23	24	Retrognathia	seq[hg19] del(1)(q21.1q21.2)chr1:g.146500000_149240000del	2.74	46, XY	Paternal	Birth
				dup(5)(p15.31p15.31)chr5:g.6560000_9660000dup	3.1			
				dup(16)(p13.11p13.11)chr16:g.15040000_16300000dup	1.26			
4	20	23 + 5	Low-lying conus medullaris, hyperechogenic spinal canal, sacral vertebrae, irregular caudal segment of the sacral vertebra, slightly hyperechoic at the left choroid plexus, cystic echo of posterior cranial fossa	seq[hg19] del(1)(q21.1q21.2)chr1:g.146500000_147840000del	1.34	46, XY	Unknown	TOP
5	35	12 + 3	Increased NT	seq[hg19] del(1)(q21.1q21.2)chr1:g.144000000_147840000del	3.84	46, XX	Maternal	Birth
6	26	22	Ectopic kidney of pelvic cavity, hyperechogenic and multiple cystic echo of renal parenchyma, absence of the gallbladder	arr[hg19] 1q21.1q21.2 (146507518–147824207) × 1	1.32	46, XY	Unknown	TOP
7	32	19	High risk of trisomy 21, pyelectasis of the right side	seq[hg19] del(1)(q21.1q21.2)chr1:g.146500000_147840000del	1.34	46, XY	Maternal	Unknown
8	41	22 + 4	Small right kidney, hyperechogenic and cystic echo of renal parenchyma	seq[hg19] dup(X)(p22.33q28)chrX:g.2710000_154930000dup	1.34	47, XXX	Unknown	Unknown
				del(1)(q21.1q21.2)chr1:g.146500000_147840000del				
9	22	23 + 3	High risk of trisomy 21, intrauterine growth retardation	seq[hg19] del(1)(q21.1q21.2)chr1:g.146500000_147840000del	1.34	46, XX	Unknown	Birth
10	32	21+	High risk of trisomy 18, bilateral choroid plexus cyst	seq[hg19] del(1)(q21.1q21.2)chr1:g.146500000_147840000del	1.34	46, XY	Unknown	Unknown
11	41	19	High risk of trisomy 21	arr[hg19] 1q21.1q21.2 (146507518–147824207) × 1	1.32	46XX, add(22)(p11.2)	*De novo*	Birth
12	29	18	High risk of trisomy 21	seq[hg19] del(1)(q21.1q21.2)chr1:g.144890000_147840000del	2.95	46, XY	Unknown	Birth
13	26	22	High risk of trisomy 18	seq[hg19] del(1)(q21.1q21.2)chr1:g.144870000_149220000del	4.35	46, XX	*De novo*	Unknown
14	35	17	Advanced maternal age	seq[hg19] del(1)(q21.1q21.2)chr1:g.146500000_149240000del	2.74	46, XX	*De novo*	Unknown
15	35	17	Advanced maternal age	seq[hg19] del(1)(q21.1q21.2)chr1:g.146500000_149240000del	2.74	46, XX	*De novo*	Unknown
16	32	21	High risk of trisomy 21	seq[hg19] del(1)(q21.1q21.2)chr1:g.146520000_147840000del	1.32	46, XX	*De novo*	TOP
17	33	11 + 4	Increased NT	arr[hg19] 1q21.1q21.2 (146507518_147824207) × 1	1.32	46, XX	Unknown	Unknown
18	31	20	High risk of trisomy 21	seq[hg19] del(1)(q21.1q21.2)chr1:g.144000000_147840000del	3.84	46, XY	Paternal	Birth
19	26	19	High risk of trisomy 21	seq[hg19] del(1)(q21.1q21.2)chr1:g.146500000_147840000del	1.34	46, XX	*De novo*	Unknown
20	30	19	High risk of trisomy 21	seq[hg19] del(1)(q21.1q21.2)chr1:g.146500000_147840000del	1.34	46, XY	Unknown	TOP
Fetuses reported in the literatures								
Zhu et al. (12)	-	-	Ventricular septal defect (VSD)	arr[hg19]1q21.1q21.2 (146043713–147929323) × 1	1.89	-	-	-
Busè et al. (13)	-	-	Bilateral cysts of the choroid plexus, a reduction in size in the ossification nucleus of the nose	[hg19] 1q21.1q21.2 (146155983_147824178) × 1	1.7	46, XY, ins(1;3)(p22;p13p23)	*De novo*	Normal birth
				[hg19] 1q21.1 (144612681_145799573) × 3	1.2			
Wang et al. (14)	26	-	Encephalomeningocele	arr[hg19] 1q21.1q21.2 (146564743–147786706) × 1	1.22	46, XN	Maternal	TOP
Wang et al. (14)	28	-	Complete atrioventricular septal defect	arr[hg19] 1q21.1q21.2 (146564743–147786706) × 1	1.22	46, XN	*De novo*	TOP
Chen et al. (15)	30	22	Polydactyly of left foot	arr[hg19]1q21.1q21.2 (146507518–147824207) × 1	1.317	46, XX.	Paternal	Birth normal
Chen et al. (16)	37	17	Oligohydramnios and bilateral renal dysplasia	arr[hg19]1q21.1q21.2 (146507518–148545520) × 1	2.038	46, XX	Maternal	TOP
Duan et al. (17)	-	-	Mild ventriculomegaly	arr[hg19]1q21.1q21.2 (146023922–147885600) × 1	1.86	-	Inherited	Birth normal
Shi et al. (18)	30	25	Transposition of the great arteries (TGA) and pulmonary valve stenosis (PVS), ventricular septal defect (VSD)	[hg19] 1q21.1q21.2 (145070868–148661862) × 1	3.59	46, XX	Maternal	TOP
Shi et al. (18)	30	25	No abnormal findings	[hg19] 1q21.1q21.2 (144877396–148636756) × 1	3.76	46, XX	Maternal	Intrauterine fetal death
Wen et al. (19)	29	21w2d	Fetal intestinal echo enhanced; high value of fetal umbilical artery blood flow spectrum S/D (the diastolic phase of blood	arr[hg19] 1q21.1q21.2 (144603950–148520164) × 1	3.9	46, XX	*De novo*	TOP
			Flow spectrum disappeared or reversed occasionally); low value of fetal ultrasound measurement; low volume of placenta size					
Wen et al. (19)	20	17w2d	No abnormality identified	seq[hg19]1q21.1q21.2 (146500001–147820000) del	1.32	46, XY	Unknown	Normal till now (3 months
Wen et al. (19)	33	18w1d	No abnormality identified	arr[hg19] 1q21.1q21.2 (146488130–147830830) × 1	1.34	46, XX	Paternal	Normal till now (10 months)
Wen et al. (19)	30	25w4d	Ventricular septal defect; racket placenta	seq[hg19]1q21.1q21.2 (146460001–147910000) del	1.45	46, XY	*De novo*	TOP
Wen et al. (19)	35	19w1d	Nuchal translucency thickness (0.45 cm)	seq[hg19]1q21.1q21.2 (146460001–147860000) del	1.4	46, XX	*De novo*	TOP
Wen et al. (19)	31	17w6d	No abnormality identified	seq[hg19]1q21.1q21.2 (146520001–147840000) del	1.32	46, XX	*De novo*	TOP
Wen et al. (19)	21	17w6d	No abnormality identified	seq[hg19]1q21.1q21.2 (144000001–147840000) del	3.84	46, XY	Maternal	Continued
Wen et al. (19)	27	18w2d	No abnormality identified	arr[hg19] 1q21.1q21.2 (145747846–147830830) × 1	2.09	46, XX	Paternal	Continued
Wen et al. (19)	20	22w5d	Narrow septum pellucidum; left lateral ventricle dysplasia	arr[hg19] 1q21.1q21.2 (145895747–147885600) × 1	1.9	46, XX	*De novo*	TOP
Wen et al. (19)	34	20w3d	No abnormality identified	seq[hg19]1q21.1q21.2 (146053252–147898839) del	1.85	46, XX	*De novo*	Continued
Su et al. (20)	27–34	23–25	Ectopic kidney (1), multicystic dysplastic kidney (1)	1q21.1q21.2 (146400000–147800000) × 1	1.3	-	Inherited/unknown	Live birth (2)
Yue et al. (21)	20	19+	Mother:1q21.1 deletion carrier with optic atrophy and retinal detachment	[hg38] 1q21.1q21.2 (147032846–148413447) × 1	1.38	46, XN	Maternal	TOP
Yue et al. (21)	31	16+	NIPT infers a high risk of chromosome 16; fetal growth restriction and microcephaly (35w 6d)	[hg38] 1q21.1q21.2 (146174424–148358701) × 1	2.63	46, XN	Maternal	Birth
Yue et al. (21)	31	18+	Increased NT(3.1 mm), abnormal childbearing history (boy: developmentally delay and intellectual disability presenting 46, XY, t(1;6) (p22;q21) and 1q21.1 microdeletion)	[hg38] 1q21.1q21.2 (146209793–148413447) × 1	2.2	46, XN	*De novo*	TOP
Yue et al. (21)	26	17+	NIPT infers a high risk of chromosome 9	[hg38] 1q21.1q21.2 (146242158–148731429) × 1	2.48	46, XN	Unknown	Birth
Yue et al. (21)	28	29+	Aberrant right subclavian artery; ventricular apical thin point	[hg38] 1q21.1q21.2 (146242158–148731429) × 1	2.47	46, XN	Unknown	Birth
Yue et al. (21)	34	18+	Risk of fetal trisomy21: 1/53	[hg38] 1q21.1q21.2 (145568752–148358701) × 1	1.8	46, XN	Unknown	TOP
				16q23.1 (75491006–75655382) × 3	0.16			
Yue et al. (21)	41	18+	Advanced maternal age	[hg38] 1q21.1q21.2 (145430996–148358701) × 1	1.93	46, XN	*De novo*	Birth
				16p11.2 (32513443–34061205) × 3	1.33			
Yue et al. (21)	30	18+	Abnormal childbearing history (a fetus presenting VSD with maternally inherited 1q21.1 deletion (TOP at 37w)	[hg38] 1q21.1q21.2 (145430996–148413447) × 1	1.98	46, XN	Maternal	Birth
Yue et al. (21)	42	22+	Advanced maternal age, increased NT	[hg38] 1q21.1 (145601946–149194711) × 1	1.39	46, XN	Maternal	Birth
Yue et al. (21)	18	28+	NIPT infers a high risk of chromosome 1	[hg38] 1q21.1q21.2 (145264933–149704737) × 1	4.43	46, XN	Maternal	TOP

CNV, copy number variation; NT, nuchal translucency; TOP, termination of pregnancy.

**Table 2:** Clinical data of all the reported fetuses with 1q21.1 microduplication syndrome.

Case	Maternal age(years)	Gestational weeks	Indications for prenatal diagnosis	CNV results	Size (Mb)	Fetal karyotype	Inheritance	Pregnancy outcome
Present cases								
21	26	14	Increased NT (8.4 mm), cervical cystic hygroma, blurred nasal bone	arr[hg19] 1q21.1q21.2 (146347096_147824207) × 3	1.48	46, XX	Unknown	TOP
22	27	30 + 5	Left lateral ventricle dilatation (left 11 mm, right 8 mm), dilated posterior horn of one lateral ventricle	arr[hg19] 1q21.1q21.2 (146347096_147824207) × 3	1.48	46, XX	Paternal	TOP
23	41	26 + 5	Mild tricuspid regurgitation, aberrant right subclavian artery	seq[hg19] dup(1)(q21.1q21.2)chr1:g.146500000_147840000dup	1.34	46, XX	Maternal	Unknown
24	24	24 + 3	Abnormal nasal bone	arr[hg19] 1q21.1q21.2 (146347096_147824207) × 3	1.48	46, XX	Paternal	Unknown
25	20	22+	L-shaped crossed fused renal ectopia, low-lying conus medullaris, abnormal vertebral formation and segmentation	arr[hg19] 1q21.1q21.2 (145415190_147824207) × 3	2.41	46, XY	Unknown	TOP
26	30	22+	Right aortic arch, aberrant right subclavian artery	arr[hg19] 1q21.11q21.2 (146507518_147824207) × 3	1.32	46, XY	Unknown	TOP
27	30	12+	Abnormal childbearing history (a fetus presenting tetralogy of Fallot at 24 weeks with paternal inherited 1q21.1 duplication syndrome (TOP))	arr[hg19] 1q21.1q21.2 (146347096_147824207) × 3	1.48	46, XX	Paternal (bicuspid aortic valve)	Unknown
28	30	23	Abnormal branching pattern of the aortic arch, polyhydramnios	arr[hg19] 1q21.1q21.2 (146347096_147824207) × 3	1.48	46, XY	Paternal	Unknown
29	34	22	Small nasal bone	arr[hg19] 1q21.1q21.2 (146347096_147824207) × 3	1.48	46, XY	*De novo*	Continued
30	38	23	Advanced maternal age, abnormal childbearing history (a girl with mental retardation)	arr[hg19] 1q21.1q21.2 (146347096_147721869) × 3	1.38	46, XY	Maternal	Unknown
31	27	19 + 6	High risk of trisomy 21	arr[hg19] 1q21.1q21.2 (146347096_147824207) × 3	1.48	46, XX	Maternal	TOP
32	25	20 + 2	High risk of neural tube defects (6.1 mm)	arr[hg19] 1q21.1q21.2 (146347096_147824207) × 3	1.48	46, XY	Maternal (immobilized joints)	Unknown
33	31	16 + 4	High risk of trisomy 21,	arr[hg19] 1q21.1q21.2 (146347096_147824207) × 3	1.48	46, XX	Maternal	Unknown
34	27	22 + 1	High risk of trisomy 21 (1/230)	seq[hg19] dup(1)(q21.1q21.2)chr1:g.146520000_147840000dup	1.32	46, XX	Paternal	Unknown
				dup(15)(q21.3q21.3)chr15:g.54640000_55920000dup				
35	28	14+	Increased NT(3.0 mm)	seq[hg19] dup(1)(q21.1q21.2)chr1:g.146500000_147840000dup	1.34	46, XY	Maternal	Birth
36	33	22 + 5	Abnormal childbearing history (a child with epilepsy)	seq[hg19] dup(1)(q21.1q21.2)chr1:g.146520000_147840000dup	1.32	46, XY	Unknown	Unknown
37	26	18	High risk of trisomy 21 (1/89)	seq[hg19] dup(1)(q21.1q21.2)chr1:g.146500000_147840000dup	1.34	46, XY	Maternal	Unknown
38	28	18	High risk of trisomy 21	seq[hg19] dup(1)(q21.1q21.2)chr1:g.144000000_147840000dup	3.84	46, XY	Unknown	Birth
39	41	19 + 2	Advanced maternal age	seq[hg19] dup(1)(q21.1q21.2)chr1:g.146500000_147840000dup	1.34	46, XX	Maternal	Birth
				dup(7)(q36.3q36.3)chr7:g.156700000_157160000dup				
40	37	19	High risk of trisomy 21, advanced maternal age	seq[hg19] dup(1)(q21.1q21.2)chr1:g.146500000_147840000dup	1.34	46, XY	Paternal	Birth
41	34	18	Increased NT (3.5 mm)	seq[hg19] dup(1)(q21.1q21.2)chr1:g.146500000_147400000dup	0.9	46, XY	Paternal	Continued
42	31	20	High risk of trisomy 21	seq[hg19] dup(1)(q21.1q21.2)chr1:g.146500000_147840000dup	1.34	46, XX	Paternal	Unknown
43	23	18 + 4	High risk of trisomy 21	seq[hg19] dup(1)(q21.1q21.2)chr1:g.146520000_147840000dup	1.32	46, XX	Maternal	Continued
Fetuses reported in the literatures								
Fu. (22)	-	-	Ventricular septal defect (VSD), pulmonary stenosis, persistent left superior vena cava	[hg19] q21.1q21.2 (146105170–147814497) × 3	1.71	-	*De novo*	TOP
Ji et al. (23)	30	24	Absent fetal nasal bone, high risk of chromosome 21 (1/215).	arr[hg19]1q21.1q21.2 (146476526–147820342) × 3	1.34	Normal	*De novo*	TOP
Ji et al. (23)	28	25	Duodenal atresia	arr[hg19]1q21.1q21.2 (146476526–147826789) × 3	1.35	Normal	Maternal	TOP
Ji et al. (23)	23	26	Nasal bone absence	arr[hg19]1q21.1q21.2 (146510112–149205098) × 3	2.69	Normal	*De novo*	TOP
Zhang et al. (24)	23	25	Short nasal bone	[hg19] 1q21.1q21.2 (146023922–147820342) × 3	1.796	46, XY,	Unknown	Top
Zhang et al. (24)	-	-	Absent nasal bone, ventricular septal defect and umbilical cord circling	[hg19] 1q21.1q21.2 (146602934–147844778) × 3	1.242	46, XY	Paternal	Birth
Wen et al. (19)	39	24w3d	Left lateral ventricle slightly widening	arr[hg19] 1q21.1q21.2 (146096700–147933973) × 3	1.8	46, XY	Paternal	Normal
Wen et al. (19)	22	23w5d	Widening; MRI: ependymal cyst?	arr[hg19] 1q21.1q21.2 (146023923–147933973) × 3	1.9	46, XX	*De novo*	Normal till now
Wen et al. (19)	29	27w3d	Left side hydrocephalus, right lateral ventricle widening	seq[hg19]1q21.1q21.2 (146500001–147840000) dup	1.34	46, XY	Paternal	TOP
Yue et al. (21)	28	18+	Increased NT, absence of nasal bone	[hg38] 1q21.1q21.2 (147024824–147921222) × 3	1.36	46, XN	Paternal	TOP
				7q36.1 (152011707–152398273) × 3	0.38			
Yue et al. (21)	29	19+	Ventricular septal defect (VSD) and the absence of nasal bone	[hg38] 1q21.1q21.2 (147131352–148372635) × 3	1.24	46, XN	Paternal	Birth
Yue et al. (21)	35	24+	Advanced maternal age, abnormal childbearing history (trisomy 21)	[hg38] 1q21.1q21.2 (147114667–148342369) × 3	1.22	46, XN	Maternal	Birth
Yue et al. (21)	26	17+	Voluntary request, no abnormal ultrasound findings observed	[hg38] 1q21.1q21.2 (147132973–148358701) × 3	1.22	46, XN	*de novo*	TOP
Yue et al. (21)	34	28+	Cerebral ventriculomegaly	[hg38] 1q21.1q21.2 (147111142–148358701) × 3	1.24	46, XN	Paternal	TOP
Yue et al. (21)	28	18+	Father: 46,XY, inv(6) (p21.1q25)	[hg38] 1q21.1q21.2 (147056729–148358701) × 3	1.30	46, XN, inv(6) (p21.1q25)	*De novo*	TOP
Yue et al. (21)	37	19+	Advanced maternal age	[hg38] 1q21.1q21.2 (147016573–148358701) × 3	1.34	46, XN	Paternal	Birth
Yue et al. (21)	33	20+	Increased NT	[hg38] 1q21.1q21.2 (147115536–148742984) × 3	1.62	46, XN	Paternal	Birth
Yue et al. (21)	35	19+	Advanced maternal age, abnormal childbearing history (child presenting cerebral palsy, developmental delay, and scoliosis)	[hg38] 1q21.1q21.2 (146066001–147919795) × 3	1.28	46, XN	Unknown	TOP
Yue et al. (21)	45	24+	Advanced maternal age, tetralogy of fallot	[hg38] 1q21.1q21.2 (146234373–148456994) × 4	2.22	46, XN	Unknown	Birth
Yue et al. (21)	23	17+	Short nasal bone	[hg38] 1q21.1q21.2 (145568752–148348214) × 3	1.79	46, XN	Unknown	TOP
				8p23.3(208048–1410532) × 1	1.2			

CNV, copy number variation; NT, nuchal translucency; TOP, termination of pregnancy.

### Cytogenetic and molecular genetic analyses

Amniotic fluid, chorionic villus, or umbilical cord blood samples were obtained according to the gestational week. Peripheral blood samples from the parents were also collected from patients who required segregation analysis. G banding karyotype analysis was performed for all amniotic fluid and umbilical cord blood samples after cell culture. The genomic DNA of the clinical samples was extracted with a Qiagen genomic DNA extraction kit. DNA concentration and purity were then detected by a NanoDrop 2000 UV‒Vis spectrophotometer (Thermo). Array comparative genomic hybridization (array CGH) analysis was performed using 8 × 60 K Agilent Oligo arrays, while copy number variation sequencing (CNV-seq) was performed via the NextSeq CN500 platform of Berry Genomics. All the patients underwent short tandem repeat (STR) analysis with a GoldeneyeTM DNA identification system kit (Peoplespot) to test for maternal contamination.

## Results

### Intrauterine phenotypes of 1q21.1 deletion syndrome

**Figure 1:**
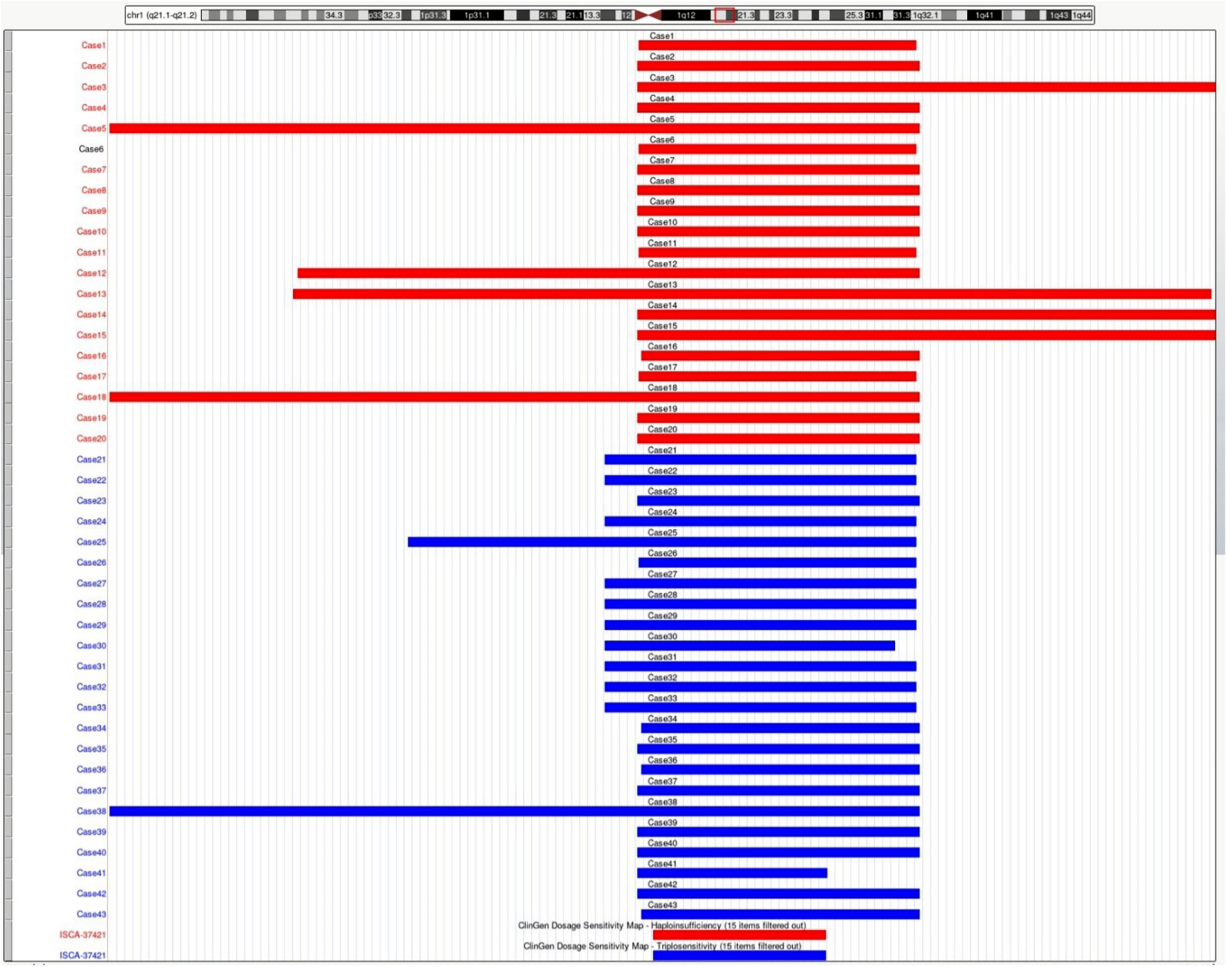
Schematic representation of the deleted or duplicated fragments in the present cases. Genomic parameters are from GRCh38/hg38.

In our study, twenty fetuses were diagnosed with 1q21.1 deletion syndrome (Table 1, Figure 1). Eleven fetuses (11/20, 55%) had abnormal ultrasound findings, while the remaining nine fetuses (9/20, 45%) were referred for prenatal diagnosis only due to a high risk of age (2/20, 10%) or serological screening (7/20, 35%). The prenatal ultrasound features mainly encompassed renal anomalies (3/20, 15%), musculoskeletal abnormalities (3/20, 15%), increased NT (3/20, 15%), neurologic abnormalities (2/20, 10%), and cardiovascular defects (1/20, 5%). Renal anomalies mainly included hyperechogenic and cystic echoes of the renal parenchyma (2/20, 10%), ectopic kidney (1/20, 5%), pyelectasis (1/20, 5%), and small kidney (1/20, 5%). Musculoskeletal abnormalities in our study included the absence of a fetal humerus and abnormal morphology of the radius, ulna, and hand in Case 1; retrognathia in Case 3; and an irregular caudal segment of the sacral vertebra in Case 4. The neurologic abnormalities included bilateral choroid plexus cysts in Case 10, a low-lying conus medullaris, hyperechoic choroid plexus and spinal canal and cystic echo of the posterior cranial fossa in Case 4. Cardiovascular defects included ventricular septal defects and coarctation of the aortic arch in Case 2. Other rare ultrasonic anomalies included the absence of the gallbladder in Case 6, intrauterine growth retardation in Case 9 and cervical cystic hygroma in Case 1.

### Intrauterine phenotypes of 1q21.1 duplication syndrome

A total of twenty-three fetuses were diagnosed with 1q21.1 duplication syndrome (Table 2, Figure 1), among which ten presented with abnormal ultrasound findings (10/23, 43.5%). The most prevalent abnormalities identified by prenatal ultrasonography included nasal bone abnormalities (3/23, 13%), cardiovascular defects (3/23, 13%), increased NT (3/23, 13%), and neurologic abnormalities (2/23, 8.7%). Cardiovascular defects contained aberrant right subclavian artery in Case 23 and Case 26, mild tricuspid regurgitation in Case 23, a right aortic arch in Case 26, and an abnormal branching pattern of the aortic arch in Case 28. Neurologic abnormalities included left lateral ventricle dilatation, dilated posterior horn of one lateral ventricle in Case 22, and low-lying conus medullaris in Case 25. Other rare ultrasonic anomalies included abnormal vertebral formation and segmentation and L-shaped crossed fused renal ectopia in Case 25, cervical cystic hygroma in Case 21, and polyhydramnios in Case 28. The remaining thirteen fetuses showed no structural anomalies, and the prenatal diagnosis reasons included a high risk of serological screening (8/23, 34.8%), advanced maternal age (3/23, 13%), and abnormal childbearing history (2/23, 8.7%).

### Cytogenetic and molecular analyses

All 43 fetuses underwent G-banded karyotype analysis, except for Case 1, 5, 21, and 35. Two abnormal karyotypes were found, namely, 47, XXX in Case 8 and 46, XX, add (24) (p11.2) in Case 11. All fetuses in our study had a common deletion or duplication in the 1q21.1 recurrent region, ranging from 0.9 Mb–4.35 Mb (Figure 1). CNV analysis also revealed an additional 3.10 Mb duplication of 5p15.31 and a 1.26 Mb duplication of 16p13.11p13.11 in Case 3 and a 1.28 Mb duplication of 15q21.3 in Case 34, all of which were of uncertain significance.

Parental analysis of the 1q21.1 region revealed that the deletions occurred *de novo* in four cases and were inherent in four cases, while duplications were inherent in 15 cases. In addition, the symptomatic father of Case 27 was described with a bicuspid aortic valve. The mother of Case 32 showed immobilized joints in both wrists, legs, and feet. The segregation was not completed due to unavailable parent samples among the remaining 20 cases.

### Follow-up

The follow-up revealed that ten fetuses continued pregnancy, including six (Case 3, 5, 9, 11, 12, and 18) with 1q21.1 deletion syndrome and four (Case 35, 38, 39, and 40) with 1q21.1 duplication syndrome. The children were delivered at gestational weeks ranging from 35–40, and the mean birth weight was 3.4 kg (Table 3). Furthermore, deletions or duplications in six children were inherited from normal parents, while one was *de novo*. The oldest child is currently three years and four months old, while the youngest is nine months old. Case 11 and Case 12 have already attended kindergarten. All the children could speak and walk at the normal stage. No abnormal findings related to growth and development, intelligence, communication, or social interaction existed. However, Case 18 was diagnosed with megacolon at two months after birth and underwent surgery at four months. In addition, Case 11 and Case 39 seem more intelligent than the siblings or peer group based on the parents' statements.

**Table 3:** Clinical data of 10 children that were born in present cases.

Case	Age of child	Childbirth	Birth weight	Age for verbal language	Age for walking	Growth and development	Intelligence	Communication and social interaction	Parental phenotype	Others
3	3 years and 2 months	Delivery by CS at 40w	3 kg	6 months	Over 1 year old	Normal	Normal	Normal	Normal	
5	2 years and 3 months	vaginal delivery at term	3.6 kg	Before 1 year	1 year and 6 months	Normal	Normal	Normal	Normal	
9	9 months	Delivery by CS at 35w	3.2 kg	-	Walking with support	Normal	Normal	Normal	Normal	
11	3 years and 4 months	Delivery by CS at term	4.5 kg	Almost 1 year old	Over 1 year old	Normal	Normal	Normal	Normal	
12	3 years and 4 months	Delivery by CS at term	3.4 kg	Around 1 year old	Over 1 year old	Normal	Normal	Normal	Normal	
18	2 years and 2 months	vaginal delivery at 38w	3.8 kg	Over 1 year old	Over 1 year old	Normal	Normal	Normal	Normal	Megacolon at 2 months old
35	1 year and 1 month	vaginal delivery at 38w6d	3.2 kg	10 months	Over 1 year old	Normal	Normal	Normal	Normal	
38	2 years and 9 months	Delivery by CS at 36w4d	2.3 kg	1 year and 6 months	1 year and 7 months	Normal	Normal	Normal	Normal	
39	2 years and 8 months	Delivery by CS at 37w6d	3.5 kg	Around 1 year old	Over 1 year old	Normal	Normal	Normal	Normal	
40	1 year and 5 months	Delivery by CS at 39w3d	3.9 kg	Around 1 year old	1 year and 1 month	Normal	Normal	Normal	Normal	

CS, cesarean section.

Six pregnancies (Case 1, 2, 4, 6, 16, and 20) with 1q21.1 deletion and five (Case 21, 22, 25, 26, and 31) with 1q21.1 duplication were selectively aborted. The remaining patients were lost to follow-up due to a telephone outage or call rejection.

## Discussion

In our study, 20 fetuses with 1q21.1 deletion syndrome and 23 fetuses with 1q21.1 duplication syndrome were described with detailed prenatal manifestations. The prenatal phenotypes of both 1q21.1 deletion and duplication syndrome fetuses were variable in the family series, ranging from a normal phenotype to musculoskeletal system abnormalities, cardiovascular defects, abnormal nervous system, and renal anomalies. The CNVs of most cases in our study were clustered into Class I deletions and duplications with only the distal region of the 1q21.1 band, while CNVs of only five cases (Case 5, 12, 13, 18, 38) were divided into Class II deletions and duplications with both the proximal and distal region of the 1q21.1 band (Figure 1). Despite the larger deletion or duplication region in the five Class II CNVs, the prenatal phenotype of the fetuses only contained increased NT and high risk of trisomy 21 or 18 without other abnormal ultrasonic findings (Tables 1, 2). It indicated that the severity of prenatal clinical phenotype might not be proportional to the CNVs size.

Most structural malformations in our study were reported prenatally for the first time and were consistent with those reported in the postnatal literature. The ultrasound findings, including retrognathia and abnormal morphology of the hand, described in Case 3 and Case 1, respectively, were once observed in a 27-year-old female patient (5). Additionally, a spinal anomaly, such as an irregular caudal segment of the sacral vertebra of Case 4, was also postnatally described as scoliosis by Digilio et al. (6). Case 7 had pyelectasis, while Case 8 had a small right kidney, which was consistent with two patients with hydronephrosis (9) and three affected patients with renal asymmetry (8). The ultrasound images of Case 23 showed tricuspid regurgitation, which had been reported in an infant (7). In contrast, some clinical phenotypes have never been reported in the literature, including the absence of fetal humerus and abnormal morphology of the radius and ulna in Case 1 and the absence of the gallbladder in Case 6.

To date, most research on 1q21.1 deletion and duplication syndrome has focused on postnatal cases. Prenatal diagnosis and genetic counseling of 1q21.1 recurrent region syndrome remain difficult due to the lack of consistent and complete prenatal phenotype features. To the best of our knowledge, only 31 fetuses with 1q21.1 deletion syndrome have been reported in the literature (Table 1), while 20 fetuses with 1q21.1 duplication syndrome have been reported (Table 2). To make a clear diagnosis of 1q21.1 deletion and duplication syndrome in the prenatal clinical phenotype, we summarize the prenatal ultrasound phenotypes of all the fetuses reported in the literature that cover the whole 1q21.1 recurrent deletion and duplication region combined with our present cases.

Fetuses with 1q21.1 deletion syndrome in our cases and the literature (Table 1, 4) commonly manifested cardiovascular defects (7/51, 13.7%), neurologic abnormalities (7/51, 13.7%), renal anomalies (6/51, 11.8%), increased NT (6/51, 11.8%), musculoskeletal abnormalities (5/51, 9.8%) and other rare phenotypes, including intrauterine growth retardation (2/51, 3.9%), placental abnormalities (2/51, 3.9%), absence of the gallbladder (1/51, 1.9%), cervical cystic hygroma (1/51, 1.9%), oligohydramnios (1/51, 1.9%), and fetal intestinal echo enhanced (1/51, 1.9%). The most common prenatal phenotype of cardiovascular defects was VSD, which was observed in 7.8% (4/51), followed by AVSD (1/51, 1.9%), PVS (1/51, 1.9%), TGA (1/51, 1.9%), ARSA (1/51, 1.9%), coarctation of the aortic arch (1/51, 1.9%), high value of s/d (1/51, 1.9%) and ventricular apical thin point (1/51, 1.9%). The main neurologic abnormalities of fetuses with 1q11.23 deletion syndrome described thus far included bilateral choroid plexus cysts (2/51, 3.9%), low-lying conus medullaris (1/51, 1.9%), hyperechogenic spinal canal (1/51, 1.9%), hyperechoic choroid plexus (1/51, 1.9%), cystic echo of posterior cranial fossa (1/51, 1.9%), encephalomeningocele (1/51, 1.9%), ventriculomegaly (1/51, 1.9%), narrow septum pellucidum (1/51, 1.9%), lateral ventricle dysplasia (1/51, 1.9%) and microcephaly (1/51, 1.9%) (12–21).

**Table 4:** Summarization of the prenatal ultrasound phenotype of all reported fetuses with 1q21.1 deletion syndrome.

Prenatal ultrasound Phenotype (total number)	Present study (total 20)	Literature reports (total 31)
Cardiovascular defects (total 7)	**1**	**6**
Ventricular septal defect (VSD)	1	3
Atrioventricular septal defect (AVSD)		1
Coarctation of aortic arch	1	
Pulmonary valve stenosis (PVS)		1
Transposition of the great arteries (TGA)		1
High value of fetal umbilical artery blood flow spectrum S/D		1
Aberrant right subclavian artery (ARSA)		1
Ventricular apical thin point		1
Musculoskeletal abnormalities (total 5)	**3**	**2**
Absence of fetal humerus	1	
Abnormal morphology of the radius, ulna and hand	1	
Retrognathia	1	
Irregular caudal segment of the sacral vertebra	1	
A reduction in size in the ossification nucleus of the nose		1
Polydactyly of left foot		1
Neurologic abnormalities (total 7)	**2**	**5**
Low-lying conus medullaris	1	
Hyperechoic spinal canal	1	
Hyperechoic choroid plexus	1	
Cystic echo of posterior cranial fossa	1	
Bilateral choroid plexus cyst	1	1
Encephalomeningocele		1
Ventriculomegaly		1
Narrow septum pellucidum		1
Lateral ventricle dysplasia		1
Microcephaly		1
Renal anomalies (total 6)	**3**	**3**
Ectopic kidney of pelvic cavity	1	1
Hyperechogenic and cystic echo of renal parenchyma	1	
Pyelectasis	1	
Small right kidney	1	
Bilateral renal dysplasia		1
Multicystic dysplastic kidney		1
Increased NT (total 6)	**3**	**3**
Others (total 7)	**3**	**4**
Absence of the gallbladder	1	
Intrauterine growth retardation	1	1
Cervical cystic hygroma	1	
Oligohydramnios		1
Fetal intestinal echo enhanced		1
Low volume of placenta size		1
Racket placenta		1
Total	**11**	**18**

The bold values mean the total number of fetuses with ultrasound abnormalities in a particular category.

Based on previous case reports and our current data (Table 2, 5), fetuses with 1q21.1 duplication syndrome were associated with a broad range of prenatal ultrasonic phenotypes, with nasal bone abnormalities (10/43, 23.3%), cardiovascular defects (7/43, 16.3%), neurologic abnormalities (6/43, 13.9%) and increased NT (5/43, 11.6%) being the most frequent. Other rare ultrasonic phenotypes included L-shaped crossed-fused renal ectopia (1/43, 2.3%), polyhydramnios (1/43, 2.3%), cervical cystic hygroma (1/43, 2.3%), duodenal atresia (1/43, 2.3%) and abnormal vertebral formation and segmentation (1/43, 2.3%). Cardiovascular defects mainly included VSD (3/43, 7%), ARSA (2/43, 4.7%), TR (1/43, 2.3%), RAA (1/43, 2.3%), PS (1/43, 2.3%), PLSVC (1/43, 2.3%) and TOF (1/43, 2.3%). The most common abnormality of the nervous system was lateral ventricle widening (3/43, 7%), followed by dilation of the posterior horn of the lateral ventricle (1/43, 2.3%), low-lying conus medullaris (1/43, 2.3%), ependymal cyst (1/43, 2.3%), hydrocephalus (1/43, 2.3%), and ventriculomegaly (1/43, 2.3%) (19, 21–24).

**Table 5:** Summarization of the prenatal ultrasound phenotype of all reported fetuses with 1q21.1 duplication syndrome.

Prenatal ultrasound Phenotype (total number)	Present study (total 23)	Literature reports (total 20)
Nasal bone abnormalities (total 10)	**3**	**7**
Cardiovascular defects (total 7)	**3**	**4**
Tricuspid regurgitation (TR)	1	
Aberrant right subclavian artery (ARSA)	2	
Right aortic arch (RAA)	1	
Abnormal branching pattern of the aortic arch	1	
Ventricular septal defect (VSD)		3
Pulmonary stenosis (PS)		1
Persistent left superior vena cava (PLSVC)	1	1
Tetralogy of Fallot (TOF)		1
Neurologic abnormalities (total 6)	**2**	**4**
Lateral ventricle widening	1	2
Dilated posterior horn of lateral ventricle	1	
Low-lying conus medullaris	1	
Ependymal cyst		1
Hydrocephalus		1
Ventriculomegaly		1
Increased NT (total 5)	**3**	**2**
Others (total 4)	**3**	**1**
Abnormal vertebral formation and segmentation	1	
L-shaped crossed fused renal ectopia	1	
Polyhydramnios	1	
Cervical cystic hygroma	1	
Duodenal atresia		1
Total	**10**	**15**

The bold values mean the total number of fetuses with ultrasound abnormalities in a particular category.

According to Tables 4, 5, consistent with postnatal reports, abnormal nasal bone function was more prevalent in fetuses with 1q21.1 duplication syndrome than in those with 1q21.1 deletion syndrome. On the other hand, renal abnormalities and musculoskeletal abnormalities were more common in fetuses with 1q21.1 deletion syndrome. This result indicated that the duplication or deletion of individual genes in the 1q21.1 recurrent region may be responsible for the difference. In addition, penetrance in all the prenatal cases might be evaluated at 56.8% (29/51) for 1q21.1 deletion syndrome and 58.1% (25/43) for reciprocal 1q21.1 duplication syndrome, which are both higher than the 36.9% and 29.1% reported for deletion and duplication, respectively, by Rosenfeld et al. (25). However, the penetrance might be overestimated due to that all the cases came to our center exhibited abnormal ultrasound findings or a high risk of prenatal screen. More prenatal cases are necessary to characterize the intrauterine phenotype of 1q21.1 deletion or duplication syndrome.

In the follow-up stage, we found that all the ten fetuses that were delivered showed normal postnatal phenotype, including birth weight, age for verbal language and walking, growth and development, intelligence, communication and social interaction, which seemed to be contrary to the well-defined postnatal phenotypes of 1q21.1 deletion or duplication syndrome in the literatures. Further analysis revealed that six out of the ten fetuses (Case 11, 12, 18, 38, 39, 40) were referred to prenatal diagnosis only due to high risk of trisomy 21 or advanced maternal age, without any ultrasonic anomaly during the pregnancy. Although the remaining four cases exhibited ultrasonic anomaly, including two fetuses (Case 5, 35) with increased NT in the first trimester, one (case 9) with intrauterine growth retardation, however, only one (Case 3) exhibited structural anomaly -retrognathia. Parental analysis revealed that deletions or duplications in six cases (Case 3, 5, 18, 35, 39, 40) were inherited from normal parents, while one (Case 11) was *de novo*. These findings may provide important cue for genetic counselors and pregnant women when considering the pregnancy outcome of fetuses with 1q21.1 deletion or duplication syndrome, especially when there were parental inheritance and no abnormal ultrasonic findings prenatally.

One major limitation of our case series is that not all cases underwent parental analysis, which may attenuate the genotype and phenotype correlation, especially when considering the incomplete penetrance and variable expressivity. The other limitation is that the pregnancy outcomes of about half of the cases were unavailable, leading to inadequate analysis of postnatal outcomes.

## Conclusion

In summary, the prenatal ultrasound findings of both 1q21.1 deletion and duplication syndrome were highly variable. Prenatal ultrasound findings, including cardiovascular defects, neurologic abnormalities, and increased NT were commonly associated with both 1q21.1 deletion and duplication syndrome. However, nasal bone abnormalities were the most common intrauterine phenotype of 1q21.1 duplication syndrome, while 1q21.1 deletion syndrome was more frequently associated with renal anomalies and musculoskeletal abnormalities. Our case series might expand the prenatal phenotype of 1q21.1 deletion and duplication syndrome by a series of new ultrasound features. Our findings provided cue insights into the genotype and prenatal phenotype correlation of 1q21.1 deletion and duplication syndrome through summarizing the prenatal phenotypes, which might benefit prenatal diagnosis and genetics counseling of the recurrent 1q21.1 region. Considering phenotypic heterogeneity and incomplete penetrance, further studies are required to elucidate the prenatal phenotype and genotype correlation of the recurrent 1q21.1 region.

## Data Availability

The raw data supporting the conclusions of this article will be made available by the authors, without undue reservation.
